# Laplacian Eigenmaps Network-Based Nonlocal Means Method for MR Image Denoising

**DOI:** 10.3390/s19132918

**Published:** 2019-07-01

**Authors:** Houqiang Yu, Mingyue Ding, Xuming Zhang

**Affiliations:** 1 Department of Biomedical Engineering, School of Life Science and Technology, Ministry of Education Key Laboratory of Molecular Biophysics, Huazhong University of Science and Technology, No 1037, Luoyu Road, Wuhan 430074, China; 2 Department of Mathematics and Statistics, Hubei University of Science and Technology, No 88, Xianning Road, Xianning 437000, China

**Keywords:** MR images, Rician noise, Laplacian eigenmaps, convolutional neural network, nonlocal means

## Abstract

Magnetic resonance (MR) images are often corrupted by Rician noise which degrades the accuracy of image-based diagnosis tasks. The nonlocal means (NLM) method is a representative filter in denoising MR images due to its competitive denoising performance. However, the existing NLM methods usually exploit the gray-level information or hand-crafted features to evaluate the similarity between image patches, which is disadvantageous for preserving the image details while smoothing out noise. In this paper, an improved nonlocal means method is proposed for removing Rician noise in MR images by using the refined similarity measures. The proposed method firstly extracts the intrinsic features from the pre-denoised image using a shallow convolutional neural network named Laplacian eigenmaps network (LEPNet). Then, the extracted features are used for computing the similarity in the NLM method to produce the denoised image. Finally, the method noise of the denoised image is utilized to further improve the denoising performance. Specifically, the LEPNet model is composed of two cascaded convolutional layers and a nonlinear output layer, in which the Laplacian eigenmaps are employed to learn the filter bank in the convolutional layers and the Leaky Rectified Linear Unit activation function is used in the final output layer to output the nonlinear features. Due to the advantage of LEPNet in recovering the geometric structure of the manifold in the low-dimension space, the features extracted by this network can facilitate characterizing the self-similarity better than the existing NLM methods. Experiments have been performed on the BrainWeb phantom and the real images. Experimental results demonstrate that among several compared denoising methods, the proposed method can provide more effective noise removal and better details preservation in terms of human vision and such objective indexes as peak signal-to-noise ratio (PSNR) and structural similarity index measure (SSIM).

## 1. Introduction

In medical imaging, high-quality images play a critical role in clinical diagnosis by enabling the clinicians to determine the state of the illness based on the structural details and functional characteristics of the images. Many imaging techniques have been developed in recent decades. Among them, magnetic resonance imaging (MRI) has attracted much attention due to its advantages of high resolution, nonradiation, noninvasiveness, and high contrast with human tissues [1]. However, the MR images are inevitably corrupted by noise. It has been shown that the noise in the MR images is governed by Rician distribution [2]. Such noise may affect the clinical diagnosis by degrading the quality of images. Removing the noise in MR images remains a necessary preprocessing step for image-based analysis and diagnosis.

Rician noise is different from Gaussian noise in that it is signal-dependent. Indeed, it is a great challenge to devise the effective denoising methods to reduce Rician noise while maintaining the integrity of image details. Numerous approaches have been developed for denoising MR images. Typical examples include the anisotropic diffusion filter [3], the bilateral filter [4], the total variation minimization filter [5], the wavelet thresholding filter [6,7], the statistical approaches [8], and nonlocal means (NLM) methods [9]. Among these methods, the NLM method [10] has aroused much interest in that it explores the self-similarity of images for denoising. This method has been originally designed for Gaussian noise removal. To extend this method to remove Rician noise, Manjon et al. [11] have proposed to adapt to the Rician noise in the MR images. Following this work, many NLM-based variations [12,13,14] have been presented. Specifically, the prefiltered rotationally invariant nonlocal means (PRI-NLM) method [12] exploits the sparseness and self-similarity properties for MR image denoising. In this method, the noisy image is first preprocessed using the oracle-based discrete cosine transform (ODCT) filter based on a moving window with local DCT hard thresholding to handle Rician noise, then the prefiltered image is further processed to produce the final restored result using the NLM filter with the rotationally invariant property. Maggioni et al. [14] have proposed a competitive BM4D method to remove the Rician noise by implementing the grouping and collaborative filtering paradigm.

Despite the advantage of the above NLM methods over the aforementioned other filters, the oversmoothing or artifact introduction is often observed in some regions denoised by these NLM methods, which may result in damage to such useful details as small objects and edges. The main reason is that these methods remove the noise based on the pixel-intensities-based similarity computation. However, the utilization of pixel intensities is not enough to characterize the self-similarity of MR images, especially at high noise corruption. To address this problem, an alternative approach is to employ the low-level features or hand-crafted features for similarity computation. The well-known hand-crafted feature descriptors include Haar-like, local binary patterns (LBP), HOG (histogram of oriented gradient), and SIFT (scale-invariant feature transform) [15,16]. Although these descriptors have succeeded in some tasks, they lack robustness and may be unable to represent the complicated features in MR images effectively [17,18]. If the robust intrinsic features can be extracted automatically from the data using the machine learning methods, it will be preferable to use these instead of pixel intensities or hand-crafted features for self-similarity computation in the NLM method to improve its denoising performance.

The deep learning, as a popular machine learning method, has been widely used for image feature extraction. Many deep learning models, such as deep belief network (DBN) [19], stacked auto-encode (SAE) [20], convolutional neural networks (CNN) [21], and principal component analysis network (PCANet) [22], have been developed and successfully applied to various visual tasks. Among these networks, the PCANet model, as a simple deep learning baseline, has attracted considerable attention. This model has shown the strong feature learning ability only through three very basic data processing components including PCA convolutional filters, binary hashing, and histograms. Compared with the CNN models which require complicated architectures design and parameters setting, the training of PCANet is very easy and efficient in that it uses the PCA to learn the convolutional kernel in an unsupervised way without requiring numerical optimization solvers or any regularization parameters.

Nevertheless, the PCA, as a linear dimension reduction method, may be improper for learning the convolutional kernel effectively for MR images which involve complicated nonlinearity. A more suitable algorithm is the Laplacian eigenmaps (LEP). As a classical nonlinear dimensionality reduction technique in the area of manifold learning [23], the LEP method is based on the spectral graph theory and its goal is to preserve the inherent manifold structure of data. Using the notion of Laplacian graph, the algorithm computes the low-dimensional representation of data by optimizing the preservation of local neighborhood information. Therefore, the neighboring data points connected in the high-dimensional space are still neighbors in the low-dimensional space after dimensionality reduction. Due to the effectiveness in mapping the data within the high-dimensional space into a low-dimensional manifold, the LEP is better for discovering nonlinear features and manifold structure embedded in the set of data [24] than such linear dimensionality reduction methods as PCA [25], multidimensional scaling [26], and linear discriminant analysis [27].

In order to extract structural features from an input MR image for the denoising task, we have proposed an LEP network (LEPNet) by emulating the architecture of PCANet. The proposed LEPNet consists of three processing layers: the two LEP-based convolutional layers and the output layer. Different from the binary-hashing-based output layer in PCANet, the LEPNet uses the activation function Leaky Rectified Linear Unit (LeakyReLU) [28,29] as the final output layer to map nonlinearity into the data. The proposed LEPNet will be used to extract the features from the pre-denoised version of the input noisy MR images and the produced features are then utilized to refine the computation of similarity weights between image patches for NLM denoising of MR images. The method noise of the denoised image is utilized to produce the final restored image. To evaluate the denoising performance of the proposed method which combines the LEPNet with the NLM filter, we have conducted extensive experiments on the simulated images and the real MR images to compare the restoration results among the proposed method and several state-of-the-art denoising methods. The visual inspection and quantitative analysis demonstrate the advantage of the proposed method in reducing noise and preserving fine details of MR images.

## 2. Methods

### 2.1. Rician Noise Model

The data of MR is a collection of the complex valued signal, whose magnitude is reconstructed to obtain the MR images. If the real and imaginary parts of this raw complex signal are corrupted by Gaussian distributed noise, the resulting MR images will have Rician distributed noise [30].

Supposing that an underlying clean MR image A is corrupted by the zero-mean Gaussian white noise with standard deviation σ in the real and imaginary channels, the noisy image $\tilde{A}$ is computed as:

(1) $$\tilde{A}=\sqrt{{\left(R+n_{R}\right)}^{2}+{\left(I+n_{I}\right)}^{2}}$$

where R and I are the real part and the imaginary part of the raw signal, respectively. $n_{R}\sim N(0,\sigma^{2})$ and $n_{I}\sim N(0,\sigma^{2})$.

Estimation of noise in MR images is a challenging task since the Rician noise is signal-dependent. To overcome the problem, Nowak [31] suggested filtering the square of the MR magnitude image, by which the noise bias in the MR image is changed to be additive and signal-independent and can be removed easily. Accordingly, we can obtain the following equation by squaring Equation (1):

(2) $$\begin{array}{cc} {\tilde{A}}^{2} & ={\left(R+n_{R}\right)}^{2}+{\left(I+n_{I}\right)}^{2}={\left(R+\sigma n_{1}\right)}^{2}+{\left(I+\sigma n_{2}\right)}^{2}\\ & =\sigma^{2}[{\left(\frac{R}{\sigma }+n_{1}\right)}^{2}+{\left(\frac{I}{\sigma }+n_{2}\right)}^{2}] \end{array}$$

where $n_{1}\sim N(0,1)$ and $n_{2}\sim N(0,1)$.

Hence, the expectation of the squared magnitude image can be calculated as:

(3) $$E({\tilde{A}}^{2})=E(A^{2})+2\sigma^{2}$$

The noise bias is equal to $2\sigma^{2}$ as indicated in Equation (3). From the image background segmented using the Otsu thresholding method [32], the noise of the MR image can be estimated as $\sigma =\sqrt{\frac{\mu }{2}}$, where μ is the mean value of the background pixels. 

### 2.2. The Proposed LEPNet Model

**Figure 1:**
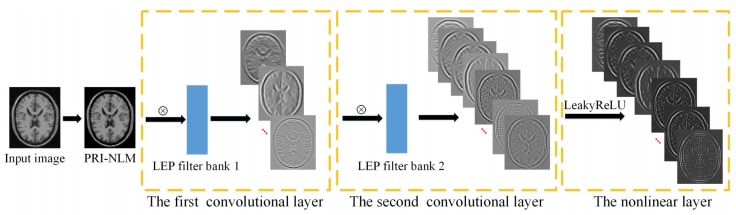
A block diagram of the proposed LEPNet model.

In this section, we will construct a LEPNet model for improving the denoising performance of the NLM method on MR images. This network includes two convolutional filter bank layers and a nonlinear processing layer. A block diagram is shown in Figure 1 to illustrate how to extract the features from an input MR image using the LEPNet. As shown in Figure 1, an image is prefiltered by the PRI-NLM method. The prefiltered image is firstly processed by the trained LEP filters to generate the feature maps in the first convolutional stage. Then, each feature map is convoluted with the trained LEP filters to produce the feature maps in the second convolutional stage. These feature maps will be processed by the following LeakyReLU function to generate the final outputs. In the LEPNet, only the LEP filters (i.e., the convolutional kernel) need to be trained from the input images. It should be noted that the LEPNet is trained on the smoothed image because the LEP is sensitive to noise and the direct application of the LEP to the input noisy image cannot produce the effective LEP filters. In what follows, the components of the LEPNet are described in detail.

#### 2.2.1. The First Convolutional Layer

To learn the convolution kernels of LEPNet, M training images of size m×n will be used. The patch size is set to be $k_{1}\times k_{2}$ at all stages. Around each pixel in the i-th input training image, the corresponding patch is collected step by step. The patch mean is then subtracted from each patch and the resulting mean-removed patch is vectorized to produce the matrix $A_{i}=[a_{i,1},a_{i,2},\cdots ,a_{i,S}]$, where $a_{i,s}$ is the s-th vector and S is the number of vectors acquired from the i-th image. All the training images are processed in the same way and put together to construct the matrix Q as:

(4) $$Q=[A_{1},A_{2},\cdots ,A_{M}]\in R^{k_{1}k_{2}\times SM}$$

The LEP operation is then implemented on Q to construct a manifold representation of data to preserve the structural information embedded in the high-dimensional space. The algorithm procedure of LEP is described below [23].

(1) Constructing the adjacency graph of Q. 

An adjacency graph on Q is defined, in which nodes $x_{p}$ and $x_{q}$ are connected by an edge if $x_{q}$ is among K-nearest-neighbor of $x_{p}$. The distance between $x_{p}$ and $x_{q}$ is determined using the Euclidean distance. 

(2) Choosing the weights.

If nodes $x_{p}$ and $x_{q}$ are connected by an edge, the weight of the edge is defined as:

(5) $$W_{p,q}=\exp(-\frac{||x_{p}-x_{q}|{|}^{2}}{t})$$

where t is the parameter of heat kernel. If $x_{p}$ and $x_{q}$ are disconnected nodes, we will set $W_{p,q}=0$. In this way, the weight matrix $W={\left(W_{pq}\right)}_{d\times d}$, a symmetric affinity matrix, can be built. 

(3) Obtaining the eigenmaps.

In order to seek the low-dimensional representation of Q, we need to minimize the cost function φ(Y) which is written as:

(6) $$\varphi (Y)=\sum_{p,q}||y_{p}-y_{q}|{|}^{2}W_{p,q}$$

where $y_{p}$ and $y_{q}$ are connected points by an edge in the low-dimensional space.

Let D denote the diagonal weight matrix whose entries are the row sums of W, thus the Laplacian matrix L is obtained by L=D−W. Accordingly, Equation (6) can be written as:

(7) $$\sum_{p,q}||y_{p}-y_{q}|{|}^{2}W_{p,q}=2tr(Z^{T}LZ)$$

where tr(·) is the trace of the matrix and $Z=[y_{1}^{T};y_{2}^{T},\cdots ;y_{M}^{T}]$ is the embedding matrix. Equation (7) is an optimization problem and its solution is the embedding matrix Z. Equation (7) can also be written as:

(8) $$\underset{Z}{\min}tr(Z^{T}LZ)\begin{array}{cc} & s.t.\begin{array}{cc} & Z^{T}DZ=I \end{array} \end{array}$$

where I is the identity matrix.

The solution of Equation (8) is sorted in an ascending order:

(9) $$\lambda_{1}\leq \lambda_{2}\leq \lambda_{3}\leq \cdots \leq \lambda_{d}$$

where d is the number of solutions of Equation (8).

Assuming that the number of filters in the first layer is $L_{1}$, the first $L_{1}$ values of Equation (9) are chosen to produce $L_{1}$ filters $O_{l}^{1}$:

(10) $$O_{l}^{1}=\underset{k_{1},k_{2}}{mat}(q_{l}(ZZ^{T}))\begin{array}{cc} & \left(l\in [1,L_{1}]\right) \end{array}$$

where $q_{l}(ZZ^{T})$ denotes the principal eigenvector corresponding to the l-th solution $\lambda_{l}$, and $\underset{k_{1},k_{2}}{mat}(q_{l}(ZZ^{T}))$ is a function for mapping $q_{l}(ZZ^{T})$ to the matrix $O_{l}^{1}$.

#### 2.2.2. The Second Convolutional Layer

To extract higher level features, the multiple stages of LEP filters will be stacked. All the outputs produced in the first convolutional layer will serve as the inputs of the second convolutional layer. By applying the same operation as the first layer, we can obtain $L_{2}$ filters of the second layer. Similarly, the process can be further repeated to capture the deeper features. In this study, we have found that the use of two convolutional layers can produce the competitive denoising performance, whereas the deeper architecture only brings little improvement.

#### 2.2.3. The Nonlinear Processing Layer

The input image will produce $L_{1}\times L_{2}$ outputs after being filtered by two convolutional layers. All these outputs will be processed to add nonlinear information to the data by using the LeakyReLU function. Here, the LeakyReLU function is defined as:

(11) $$\mathrm{LeakyReLU}(x)=\{\begin{array}{c} x\begin{array}{cc} & if\begin{array}{cc} & x\geq 0 \end{array} \end{array}\\ \frac{x}{a}\begin{array}{cc} & if\begin{array}{cc} & x<0 \end{array} \end{array} \end{array}$$

where a is a coefficient controlling the slope of the negative part. Compared with the classical activation functions such as ReLU and sigmoid, the LeakyReLU is more effective in preserving the image details in that the structural information corresponding to the negative values in the image can be maintained.

### 2.3. The LEPNet-Based Nonlocal Means Method

The similarity in the traditional NLM algorithm is determined by exploiting the gray-level information itself. Such a scheme may result in the unreliable determination of similarity weights. Therefore, we have proposed a shallow deep learning model LEPNet to extract the robust intrinsic features from the input MR image to refine the similarity weights in the NLM filter. To learn the intrinsic features by the LEPNet, we will train the model to produce the convolution kernels. Considering that the training of LEPNet is performed using the patch-based learning strategy, even a small number of images can produce a large number of image patches. Thus, we only use 300 general MR images from the open MRI database [33,34,35,36] as the training samples to train the LEPNet model. All the 300 images are cropped to 160 × 160 pixels and prefiltered by the PRI-NLM filter.

By using the trained convolution kernels, the LEPNet can capture the structural features from the input MR images. The $L(L=L_{1}\times L_{2})$ feature maps produced from two convolutional layers will be processed by the LeakyReLU function to provide the final outputs. 

In the traditional NLM (TNLM) method, each restored pixel is the weighted mean of all other pixels in a search window in the noisy image:

(12) $$NLM[\tilde{A}(i_{1},j_{1})]=\sum_{\left(i_{2},j_{2})\in \Omega (i_{1},j_{1}\right)}\tilde{A}(i_{2},j_{2})\omega (i_{1},j_{1},i_{2},j_{2})$$

where $NLM[\tilde{A}(i_{1},j_{1})]$ is the filtered intensity at location $\left(i_{1},j_{1}\right)$ in the noisy image $\tilde{A}$ by using the NLM method, $\Omega (i_{1},j_{1})$ is the search window centered at $\left(i_{1},j_{1}\right)$, and $\omega (i_{1},j_{1},i_{2},j_{2})$ is the similarity between two image patches centered at $\left(i_{1},j_{1}\right)$ and $\left(i_{2},j_{2}\right)$, which is defined as:

(13) $$\omega (i_{1},j_{1},i_{2},j_{2})=\frac{1}{Z(i_{1},j_{1})}\exp(-\frac{Dis(i_{1},j_{1},i_{2},j_{2})}{h^{2}})$$

where $Z(i_{1},j_{1})$ is the normalization constant ensuring $\sum_{\left(i_{2},j_{2})\in \Omega (i_{1},j_{1}\right)}\omega (i_{1},j_{1},i_{2},j_{2})=1$ and is defined as $Z(i_{1},j_{1})=\sum_{\left(i_{2},j_{2})\in \Omega (i_{1},j_{1}\right)}\exp(-\frac{Dis(i_{1},j_{1},i_{2},j_{2})}{h^{2}})$, $Dis(i_{1},j_{1},i_{2},j_{2})$ is the Euclidean distance computed as:

(14) $$Dis(i_{1},j_{1},i_{2},j_{2})=||\tilde{A}(i_{1},j_{1})-\tilde{A}(i_{2},j_{2})|{|}_{2,a}^{2}$$

where α is the standard deviation of Gaussian kernel function, h is the decay parameter and it is determined using the rule-of-thumb [37], that is, h=Cσ, where C denotes a constant. The rule works well in this study. To effectively determine h, the noise standard deviation σ of the MR image needs to be estimated using Equation (3).

**Figure 2:**
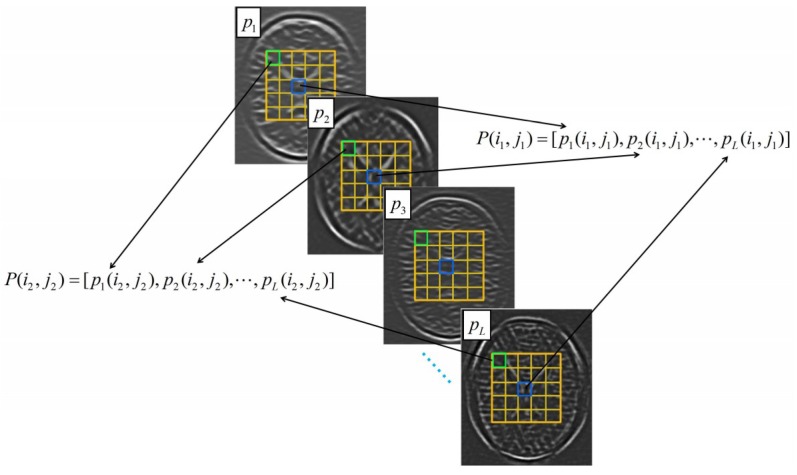
The construction of feature vectors based on the feature images of input MR images.

Instead of the utilization of gray-level patterns around each pixel, the proposed method refines the computation of similarity weight based on the feature vectors obtained by the LEPNet model. Figure 2 illustrates how to construct the feature vectors related to the pixels. The considered image patch in the noisy image is represented with a feature vector by concatenating the pixel intensities of the same location in all feature images as shown in Figure 2. Therefore, the features for the image patch centered at the pixel $\left(i_{1},j_{1}\right)$ in a search window marked by the yellow boxes can be represented as:

(15) $$P(i_{1},j_{1})=[p_{1}(i_{1},j_{1}),p_{2}(i_{1},j_{1}),\cdots ,p_{L}(i_{1},j_{1})]$$

where $p_{1}(i_{1},j_{1}),p_{2}(i_{1},j_{1}),\cdots ,p_{L}(i_{1},j_{1})$ are the pixel intensities in the feature images marked by the blue boxes in Figure 2. Likewise, the features for the image patch centered at another pixel $\left(i_{2},j_{2}\right)$ marked by the green boxes in Figure 2 can be denoted as:

(16) $$P(i_{2},j_{2})=[p_{1}(i_{2},j_{2}),p_{2}(i_{2},j_{2}),\cdots ,p_{L}(i_{2},j_{2})]$$

The structural similarity $\omega (i_{1},j_{1},i_{2},j_{2})$ between two pixels $\left(i_{1},j_{1}\right)$ and $\left(i_{2},j_{2}\right)$ is calculated using the corresponding feature vectors $P(i_{1},j_{1})$ and $P(i_{2},j_{2})$. Compared with the patch-based computation strategy in the TNLM method, the calculation of the Euclidean distance based on the constructed feature vectors can greatly improve the computational efficiency.

Considering that some image details will be lost during denoising, the residual details in the method noise [38], which is defined as the difference between the noisy image and its denoised version, will be extracted. Here the method noise is firstly filtered to generate the residual image using the NLM method where the weight $\omega (i_{1},j_{1},i_{2},j_{2})$ in Equation (13) is used. Then, a 3×3 mean filter is implemented on the residual image to smooth residual noise, thereby producing the residual detail image $r(\tilde{A}(x,y))$. As a result, the final restored image $\overset{\wedge }{A}(x,y)$ can be computed as $\overset{\wedge }{A}(x,y)=NLM(\tilde{A}(x,y))+r(\tilde{A}(x,y))$.

### 2.4. Overall Description of the Proposed Denoising Algorithm

**Figure 3:**
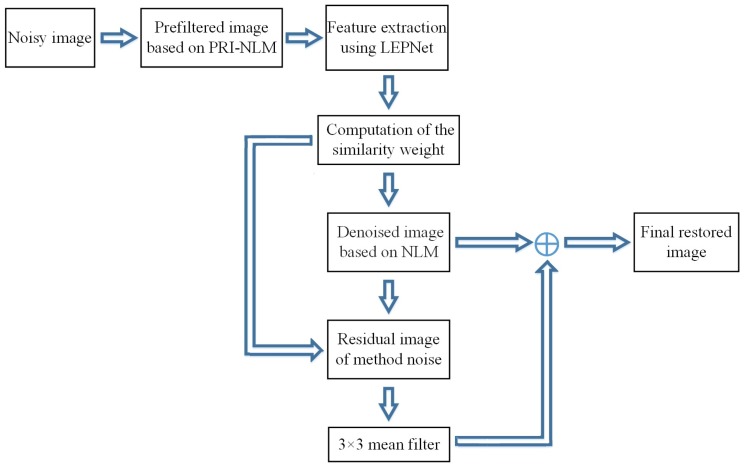
Block diagram of the proposed denoising method.

We refer to the proposed denoising method as LEP-NLM. A block diagram of the LEP-NLM method is shown in Figure 3. The implementation details of this method are summarized as follows.

Step 1: The LEPNet model is trained using 300 PRI-NLM filtered MR images obtained from the open MRI database to learn the convolution kernels of two convolutional layers.
Step 2: The input noisy MR image is preprocessed by the PRI-NLM filter to produce the pre-denoised image, then it is input into the trained LEPNet model to generate L feature images as the output of this model.
Step 3: Based on the obtained feature images, the feature vectors related to the pixels are constructed for calculating the similarity weights.
Step 4: Based on the obtained similarity weights and the decay parameter, the input MR image is denoised using the NLM algorithm to produce the denoised image.
Step 5: The method noise for the denoised image produced in Step 4 is processed by the NLM method and a 3 × 3 mean filter to retrieve the lost image details in the denoised image.
Step 6: By combining the denoised image in Step 4 and the retrieved details in Step 5, the final restored image can be obtained.

## 3. Experimental Results and Discussion

To evaluate the effectiveness of the proposed method, several experiments have been conducted on different datasets including the well-known BrainWeb phantom [39] and the real brain image dataset Atlas [40]. The performance of the LEP-NLM method is compared with that of some related MRI denoising methods, such as the Wiener filter, the TNLM filter, the wavelet sub-band coefficient mixing (WSM) filter [41], the ODCT filter [12], the PRI-NLM filter, and the BM4D filter. In all experiments, the 3 × 3 filtering window is used for the Wiener filter. For these NLM-based filters, the size of the similarity window and the search window are set to 7 × 7 and 17 × 17, respectively. Additionally, the decay parameter h is set to be proportional to the standard deviation σ of the noisy image (i.e., h=Cσ). Here, σ is estimated according to Equation (3). The constant C will influence the restoration performance. If C is too small, little noise will be removed. Otherwise, the image will be overly smoothed and some image details will be damaged. C will be determined through the visual impression and quantitative indexes. For the simulated MR images, C is chosen based on the quantitative results and the visual inspection. In the real images, C is determined only by the visual inspection since the ground truth is unavailable. The WSM, ODCT, and BM4D filters are run using the parameters as suggested by the authors. For the proposed method, a two-layer LEPNet model is used and the number of LEP filters in the convolutional layers is set to 12, the size of the image patch and convolutional kernel are both 7 × 7, and the hyperparameter a of LeakyReLU function is set to 3 through the grid search.

To evaluate all compared methods quantitatively, two widely used quantitative metrics including peak signal-to-noise ratio (PSNR) and structural similarity index measure (SSIM) are considered [42], which are computed as:

(17) $$PSNR(A(x,y),\hat{A}(x,y))=10\cdot \log_{10}{\left(\frac{255}{\sqrt{\frac{1}{A_{W}\cdot A_{H}}\sum_{x=0}^{A_{W}-1}\sum_{y=0}^{A_{H}-1}||A(x,y)-\hat{A}(x,y)|{|}^{2}}}\right)}^{2}$$

(18) $$SSIM(A(x,y),\hat{A}(x,y))=\frac{\left(2\mu_{A}\mu_{\hat{A}}+c_{1})(2\sigma_{A\hat{A}}+c_{2}\right)}{\left(\mu_{A}^{2}+\mu_{\hat{A}}^{2})(\sigma_{A}^{2}+\sigma_{\hat{A}}^{2}+c_{2}\right)}$$

where A(x,y) and $\hat{A}(x,y)$ are the noise-free image and the filtered image, respectively. $A_{W}$ and $A_{H}$ are the width and height of A(x,y), $\mu_{A}$ and $\mu_{\hat{A}}$ are the average of A(x,y) and $\hat{A}(x,y)$, $\sigma_{A}$ and $\sigma_{\hat{A}}$ are the variance of A(x,y) and $\hat{A}(x,y)$, respectively. $\sigma_{A\hat{A}}$ is the covariance of images A(x,y) and $\hat{A}(x,y)$, $c_{1}$ and $c_{2}$ are two constants of small value for stabilizing the division with a weak denominator.

In addition to visual quality, the computational complexity is an important aspect for the denoising method. In this study, all the experiments are performed on a personal computer with the Matlab (R2017a) environment, a 2.30 GHz i5 processor, and 24 GB DDR4 of RAM. We will compare the run time of the TNLM and the LEP-NLM for denoising images of size 181 × 217 and 256 × 256. For the two images, the denoising time of the TNLM is 50.48 s and 91.43 s, respectively. By comparison, the implementation time of the LEP-NLM is 23.10 s and 25.45 s, respectively. The results show that the computational efficiency of the LEP-NLM is significantly higher than that of the TNLM. 

### 3.1. Simulated MR Images

The simulated images of size 181 × 217 are taken from the BrainWeb phantom. They include T1-, T2-, and PD-weighted MR images. Each image is corrupted with various levels of simulated Rician noise. The standard deviation σ of noise is computed as σ=μ⋅max(A) where μ is the noise proportion and max(A) denotes the maximum intensity of the noise-free image A. In this paper, μ takes 2%, 5%, 10%, 15%, 20%, 25%, and 30%. 

**Figure 4:**
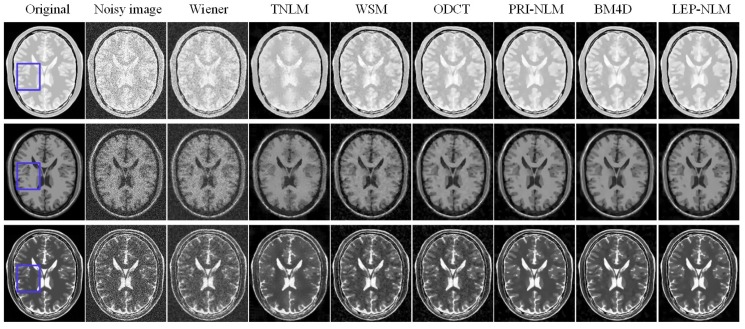
Visual inspection of the denoised results of Wiener, TNLM, WSM, ODCT, PRI-NLM, BM4D, and LEP-NLM filters on the BrainWeb phantom corrupted by the Rician noise level of 15%. The top row, middle row, and bottom row are the PD-weighted, T1-weighted, and T2-weighted MR images, respectively.

An example is given to illustrate the denoising performance among the considered filters by adding 15% Rician noise to the original image. Figure 4 depicts the visual comparison of the restored images using the different methods. Significant noise is observed in the image obtained by the Wiener filter. In contrast, the TNLM method is effective in reducing noise, but it blurs the edges and distorts the lines. The WSM and ODCT filters generate the Gibbs artifacts in the smooth region although they can preserve the sharp edges in the images. The PRI-NLM filter tends to produce the oversmoothing of image details in some regions, while the BM4D filter may cause the damage to the small structures. However, the LEP-NLM filter achieves satisfactory visual impression in that the noise is removed effectively, the artifacts are avoided, and the fine details are preserved well.

**Figure 5:**
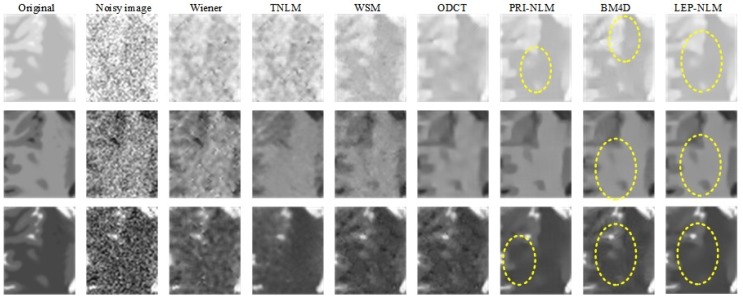
Visual comparison of denoised results for the enlarged ROIs marked with the blue boxes in Figure 4. The top row, middle row, and bottom row are the PD-weighted, T1-weighted, and T2-weighted MR images, respectively.

In order to visualize the structural information more clearly, the enlarged views of the regions of interest (ROIs) marked with the blue boxes in Figure 4 are presented in Figure 5. Here, ROIs include the cerebral gyri and sulcus. Obviously, the images produced by the Wiener, the TNLM, and the WSM filters are unsatisfactory because too much noise and artifacts remain or edges and small objects are damaged. The ODCT filter blurs the boundaries between the cerebral gyri and sulcus, while the PRI-NLM and BM4D filters tend to oversmooth the sulcus area, which leads to the loss of structural information. More specifically, we have compared the proposed method with such competitive methods as the PRI-NLM and BM4D methods. Some regions are chosen and marked with the yellow elliptic dotted lines as shown in Figure 5. In the PD-weighted image, the PRI-NLM filter generates the blurred edges while the obvious artifact can be observed for the BM4D method. In the T1-weighted image, the small objects are smoothed by the BM4D filter. In the T2-weighted image, the PRI-NLM and BM4D filters produce the similar negative influence as they have done on the PD-weighted image. By comparison, the LEP-NLM filter works well on recovering visually significant structures while removing noise and artifacts.

The PSNR and SSIM results of various methods on the PD-weighted, T1-weighted, and T2-weighted MR images are shown in Table 1, Table 2 and Table 3, respectively. Specifically, the LEP-NLM method without additionally using the method noise is denoted by the LEP-NLM_w_. The best value for each corrupted image at each noise level is represented in bold. As one can see, the LEP-NLM method always achieves the best PSNR and SSIM values at almost all noise levels except that its PSNR and SSIM values are slightly lower than those of the BM4D method for denoising PD-weighted images with 2% Rician noise and T1-weighted images with three levels of Rician noise, which demonstrates that the proposed method is more effective in suppressing Rician noise and preserving fine structures in the image. Additionally, the comparison between the LEP-NLM_w_ and the LEP-NLM shows that the PSNR and SSIM of the latter could benefit from the utilization of the method noise.

### 3.2. Real MR Images

Two real clinical MR images of size 256 × 256 are acquired from the Atlas dataset [38] for evaluating all compared methods. Here, one image is a normal sagittal T2-weighted brain MR image and another is a transaxial T1-weighted brain MR image with cerebrovascular disease. In this experiment, quantitative assessment is no longer feasible because the ground truth of the real images is unavailable. Therefore, we will perform the visual inspection on the filtered images.

**Figure 6:**
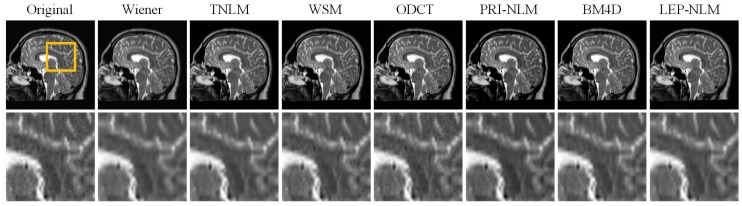
The restoration results of a real T2-weighted brain MR image by the various denoising filters. From top to bottom: the original MR images and the corresponding enlarged portions of ROIs marked with the yellow box in the original images.

**Figure 7:**
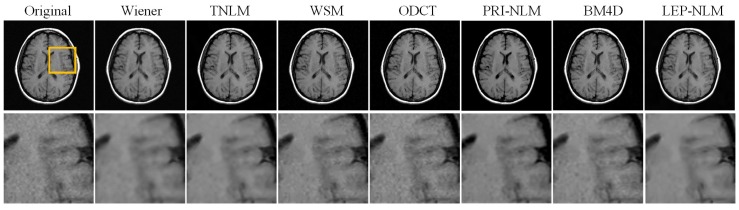
The restoration results of a real T1-weighted brain MR image by the various denoising filters. From top to bottom: the original MR images and the corresponding enlarged portions of ROIs marked with the yellow box in the original images.

Figure 6 and Figure 7 depict the denoised results of the two real MR images for the compared methods. It can be seen that the LEP-NLM method provides satisfactory restoration performance since it not only smooths out the noise but also preserves the meaningful structural details and maintains the image contrast. Furthermore, the zoomed details of two images are shown. Obviously, the Wiener method blurs the boundaries while the TNLM method produces unwanted artifacts. In the denoised images by the WSM, ODCT, and PRI-NLM methods, there exists much residual noise. For the BM4D method, the fine details have not been preserved well although the noise is removed effectively. However, the proposed method achieves better performance in structural information preservation by enhancing the edges and small objects.

## 4. Conclusions

In this paper, we have developed a novel denoising method by combining the LEPNet and the NLM method to reduce the Rician noise in MR images. To improve the accuracy of similarity weight computation, we have designed an unsupervised shallow convolutional network LEPNet to extract the image features and used them to refine similarity computation. By means of the two cascaded LEP-based convolutional layers and a LeakyReLU-based nonlinear layer, the LEPNet model can extract the robust intrinsic image features, which ensures that the self-similarity can be represented more effectively than using the pixel intensities in the existing NLM methods. The experimental results demonstrate that the proposed LEP-NLM method is very effective in removing the Rician noise and preserving the structural details of images. Therefore, the proposed method can potentially assist clinical experts in MRI-based disease diagnosis by preserving the tiny structure of lesions. Future study will be focused on investigating more deep learning networks to further improve the denoising performance of the NLM-based method.

## Figures and Tables

**Table 1:** PSNR/dB and SSIM comparison on the simulated PD-weighted images at different noise levels for the Wiener, TNLM, WSM, ODCT, PRI-NLM, BM4D, LEP-NLM, and LEP-NLM_w_ methods.

Methods	σ	Noisy	Wiener	TNLM	WSM	ODCT	PRI-NLM	BM4D	LEP-NLM_w_	LEP-NLM
PSNR	2%	33.47	33.69	39.47	38.59	42.58	43.49	**43.68**	43.53	43.58
	5%	25.40	27.76	33.80	34.03	36.60	36.98	37.31	37.83	**37.91**
	10%	19.46	22.34	29.07	29.25	31.69	31.90	32.49	32.62	**32.85**
	15%	16.12	18.93	26.38	26.07	28.71	28.94	29.61	29.66	**30.00**
	20%	13.94	16.51	24.00	23.63	26.55	26.94	27.52	27.61	**28.03**
	25%	12.19	14.65	22.38	21.68	24.72	25.28	25.85	25.96	**26.44**
	30%	10.88	13.12	20.42	20.07	23.14	23.92	24.38	24.63	**25.15**
SSIM	2%	0.780	0.868	0.976	0.969	0.977	0.984	0.982	**0.985**	**0.985**
	5%	0.558	0.786	0.936	0.904	0.945	0.950	0.946	0.952	**0.960**
	10%	0.377	0.660	0.842	0.810	0.876	0.898	0.896	0.887	**0.915**
	15%	0.285	0.544	0.761	0.718	0.817	0.848	0.851	0.832	**0.870**
	20%	0.233	0.444	0.694	0.630	0.768	0.801	0.809	0.780	**0.823**
	25%	0.191	0.377	0.624	0.552	0.726	0.761	0.766	0.738	**0.781**
	30%	0.157	0.331	0.559	0.483	0.683	0.726	0.727	0.703	**0.745**

**Table 2:** PSNR/dB and SSIM comparison on the simulated T1-weighted images at different noise levels for the Wiener, TNLM, WSM, ODCT, PRI-NLM, BM4D, LEP-NLM, and LEP-NLM_w_ methods.

Methods	σ	Noisy	Wiener	TNLM	WSM	ODCT	PRI-NLM	BM4D	LEP-NLM_w_	LEP-NLM
PSNR	2%	33.57	34.13	40.24	41.07	43.30	44.05	44.21	44.64	**44.82**
	5%	25.66	28.19	34.56	34.76	37.16	37.84	38.01	38.38	**38.64**
	10%	19.63	22.42	29.45	29.34	32.11	32.82	33.10	33.42	**33.82**
	15%	16.10	18.77	25.95	26.06	29.00	29.69	29.93	30.36	**30.85**
	20%	13.59	16.17	23.63	23.68	26.80	27.28	27.64	28.02	**28.58**
	25%	11.72	14.13	21.34	21.79	24.97	25.41	25.89	26.17	**26.73**
	30%	10.25	12.53	19.84	20.19	23.51	24.00	24.48	24.67	**25.25**
SSIM	2%	0.746	0.832	0.972	0.959	0.985	0.985	0.983	0.977	**0.987**
	5%	0.568	0.762	0.912	0.881	0.931	0.951	0.949	0.927	**0.957**
	10%	0.378	0.625	0.825	0.778	0.851	0.898	0.901	0.854	**0.903**
	15%	0.259	0.497	0.727	0.682	0.782	0.844	0.855	0.798	**0.858**
	20%	0.182	0.406	0.645	0.595	0.727	0.787	**0.809**	0.745	0.798
	25%	0.133	0.327	0.545	0.516	0.674	0.729	**0.760**	0.694	0.744
	30%	0.101	0.268	0.444	0.443	0.629	0.684	**0.711**	0.647	0.694

**Table 3:** PSNR/dB and SSIM comparison on the simulated T2-weighted images at different noise levels for the Wiener, TNLM, WSM, ODCT, PRI-NLM, BM4D, LEP-NLM, and LEP-NLM_w_ methods.

Methods	σ	Noisy	Wiener	TNLM	WSM	ODCT	PRI-NLM	BM4D	LEP-NLM_w_	LEP-NLM
PSNR	2%	33.46	35.07	37.99	38.42	42.07	42.82	43.03	**43.59**	**43.59**
	5%	25.42	26.53	32.07	33.35	35.58	36.10	36.25	36.93	**37.00**
	10%	19.30	21.64	26.70	28.13	30.54	31.06	31.28	31.79	**31.97**
	15%	15.91	18.32	23.76	24.77	27.52	27.96	28.24	28.75	**29.01**
	20%	13.54	15.73	21.94	22.23	25.14	25.57	26.08	26.40	**26.70**
	25%	11.64	13.78	19.97	20.21	22.97	23.46	24.32	24.39	**24.70**
	30%	10.10	12.05	18.43	18.59	21.11	21.66	22.83	22.66	**22.96**
SSIM	2%	0.797	0.867	0.978	0.972	0.979	0.986	0.983	0.988	**0.989**
	5%	0.620	0.792	0.938	0.911	0.952	0.956	0.952	0.957	**0.966**
	10%	0.468	0.691	0.847	0.811	0.888	0.913	0.907	0.901	**0.928**
	15%	0.375	0.592	0.755	0.718	0.830	0.867	0.854	0.851	**0.888**
	20%	0.309	0.494	0.709	0.632	0.773	0.820	0.808	0.804	**0.843**
	25%	0.247	0.415	0.604	0.552	0.708	0.767	0.764	0.760	**0.799**
	30%	0.199	0.347	0.526	0.484	0.632	0.700	0.719	0.711	**0.749**

## References

[1] Ikram S., Shah J.A., Zubair S., Qureshi I.M., Bilal M. (2019). Improved reconstruction of MR scanned images by using a dictionary learning scheme. Sensors.

[2] Sharma A., Chaurasia V. (2018). A review on magnetic resonance images denoising techniques. Mach. Intell. Signal Anal..

[3] Murase K., Yamazaki Y., Shinohara M., Kawakami K., Kikuchi K., Miki H., Ikezoe J. (2001). An anisotropic diffusion method for denoising dynamic susceptibility contrast-enhanced magnetic resonance images. Phys. Med. Biol..

[4] Ghassan H., Judith H. (2007). Bilateral filtering of diffusion tensor magnetic resonance images. IEEE Trans. Image Process..

[5] Liu R.W., Shi L., Huang W., Xu J., Yu S.C.H., Wang D. (2014). Generalized total variation-based MRI Rician denoising model with spatially adaptive regularization parameters. Magn. Reson. Imaging.

[6] Izlian Y.O., Francisco J.G., Alfonso A. (2019). Local complexity estimation based filtering method in wavelet domain for magnetic resonance imaging denoising. Entropy.

[7] Pizurica A., Wink A.M., Vansteenkiste E., Philips W., Roerdink B.J. (2006). A review of wavelet denoising in MRI and ultrasound brain imaging. Curr. Med. Imaging Rev..

[8] Zervakis M.E., Katsaggelos A.K., Kwon T.M. (1995). A class of robust entropic functionals for image restoration. IEEE Trans. Image Process..

[9] Hemalata V.B., Basavaraj H.V. (2019). NLM based magnetic resonance image denoising-A review. Biomed. Signal Process. Control.

[10] Buades A., Coll B., Morel J.M. A non-local algorithm for image denoising. Proceedings of the IEEE Computer Society Conference on Computer Vision and Pattern Recognition (CVPR).

[11] Manjón J.V., Carbonell-Caballero J., Lull J.J., García-Martí G., Martí-Bonmatí L., Robles M. (2008). MRI denoising using non-local means. Med. Image Anal..

[12] Manjon J.V., Coupe P., Buades A., Collins D.L., Robles M. (2012). New methods for MRI denoising based on sparseness and self-similarity. Med. Image Anal..

[13] Manjon J.V., Coupe P., Buades A. (2015). MRI noise estimation and denoising using non-local PCA. Med. Image Anal..

[14] Maggioni M., Katkovnik V., Egiazarian K., Foi A. (2013). Nonlocal transform-domain filter for volumetric data denoising and reconstruction. IEEE Trans. Image Process..

[15] Zhu Y., Wang L., Liu M., Qian C., Yousuf A., Oto A., Shen D. (2017). MRI-based prostate cancer detection with high-level representation and hierarchical classification. Med. Phys..

[16] Schonberger J.L., Hardmeier H., Sattler T., Pollefeys M. Comparative evaluation of hand-crafted and learned local features. Proceedings of the IEEE Computer Society Conference on Computer Vision and Pattern Recognition (CVPR).

[17] Hinton G.E., Osindero S., Teh Y.-W. (2006). A fast learning algorithm for deep belief nets. Neural Comput..

[18] Bengio Y., Courville A., Vincent P. (2013). Representation learning: A review and new perspectives. IEEE Trans. Pattern Anal. Mach. Intell..

[19] Li C., Wang Y., Zhang X., Gao H., Yang Y., Wang J. (2019). Deep belief network for spectral-spatial classification of hyperspectral remote sensor data. Sensors.

[20] Zhao F., Liu Y., Huo K., Zhang S., Zhang Z. (2018). Radar HRRP target recognition based on stacked autoencoder and extreme learning machine. Sensors.

[21] Zhang K., Zuo W., Chen Y., Meng D., Zhang L. (2017). Beyond a Gaussian denoiser: Residual learning of deep CNN for image denoising. IEEE Trans. Image Process..

[22] Chan T.H., Jia K., Gao S., Lu J., Zeng Z., Ma Y. (2015). PCANet: A simple deep learning baseline for image classification?. IEEE Trans. Image Process..

[23] Belkin M., Niyogi P. (2003). Laplacian eigenmaps for dimensionality reduction and data representation. Neural Comput..

[24] Van der Maaten L., Postma E., Van Den Herik H.J. (2009). Dimensionality reduction: A comparative review. J. Mach. Learn. Res..

[25] Daffertshofer A., Lamoth C.J., Meijer O.G., Beek P.J. (2004). PCA in studying coordination and variability: A tutorial. Clin. Biomech..

[26] Borg I., Groenen P.J.F. (2006). Modern multidimensional scaling: Theory and applications. J. Educ. Meas..

[27] Portillo-Portillo J., Leyva R., Sanchez V., Sanchez-Perez G., Perez-Meana H., Olivares-Mercado J., Nakano-Miyatake M. (2017). Cross view gait recognition using joint-direct linear discriminant analysis. Sensors.

[28] Krizhevsky A., Sutskever I., Hinton G.E. Imagenet classification with deep convolutional neural networks. Proceedings of the 25th International Conference on Neural Information Processing Systems.

[29] Andrew L.M., Awni Y.H., Andrew Y.N. Rectifier nonlinearities improve neural network acoustic models. Proceedings of the 30th International Conference on Machine Learning.

[30] Basu S., Fletcher T., Whitaker R. Rician noise removal in diffusion tensor MRI. Proceedings of the International Conference on Medical Image Computing and Computer-Assisted Intervention.

[31] Nowak R.D. (1999). Wavelet-based rician noise removal for magnetic resonance imaging. IEEE Trans. Image Process..

[32] Otsu N. (1979). A threshold selection method from gray-level histograms. IEEE Trans. Syst. Man Cybern..

[33] The Cancer Imaging Archive.

[34] Litjens G., Debats O., Barentsz J., Karssemeijer N., Huisman H. ProstateX challenge data. The Cancer Imaging Archive.

[35] Litjens G., Debats O., Barentsz J., Karssemeijer N., Huisman H. (2014). Computer-aided detection of prostate cancer in MRI. IEEE Trans. Med. Imaging.

[36] Clark K., Vendt B., Smith K., Freymann J., Kirby J., Koppel P., Tarbox L. (2013). The cancer imaging archive (TCIA): Maintaining and operating a public information repository. J. Digit. Imaging.

[37] Kervrann C., Boulanger J. (2008). Local adaptivity to variable smoothness for exemplar-based image regularization and representation. Int. J. Comput. Vis..

[38] Zhong H., Yang C., Zhang X. (2012). A new weight for nonlocal means denoising using method noise. IEEE Signal Process. Lett..

[39] BrainWeb: Simulated Brain Database. http://brainweb.bic.mni.mcgill.ca/brainweb/.

[40] The Whole Brain Atlas. http://www.med.harvard.edu/aanlib/home.html.

[41] Coupe P., Hellier P., Prima S., Kervrann C., Barillot C. (2008). 3D Wavelet Sub-Bands Mixing for Image Denoising. Int. J. Biomed. Imaging.

[42] Kala R., Deepa P. (2018). Adaptive hexagonal fuzzy hybrid filter for Rician noise removal in MRI images. Neural Comput. Appl..

